# Metformin Lysosomal Targeting: A Novel Aspect to Be Investigated for Metformin Repurposing in Neurodegenerative Diseases?

**DOI:** 10.3390/ijms25168884

**Published:** 2024-08-15

**Authors:** Nadia Papini, Paola Giussani, Cristina Tringali

**Affiliations:** Department of Medical Biotechnology and Translational Medicine, Università degli Studi di Milano, LITA Segrate, 20054 Segrate, MI, Italy; nadia.papini@unimi.it (N.P.); paola.giussani@unimi.it (P.G.)

**Keywords:** metformin, lysosome, neurodegenerative diseases

## Abstract

Metformin is a widely employed drug in type 2 diabetes. In addition to warranting good short- and long-term glycemic control, metformin displays many intriguing properties as protection against cardiovascular and neurodegenerative diseases, anti-tumorigenic and longevity promotion. In addition to being a low-cost drug, metformin is generally well tolerated. However, despite the enthusiastic drive to aliment these novel studies, many contradictory results suggest the importance of better elucidating the complexity of metformin action in different tissues/cells to establish its possible employment in neurodegenerative diseases. This review summarises recent data identifying lysosomal-dependent processes and lysosomal targets, such as endosomal Na^+^/H^+^ exchangers, presenilin enhancer 2 (PEN2), the lysosomal pathway leading to AMP-activated protein kinase (AMPK) activation, and the transcription factor EB (TFEB), modulated by metformin. Lysosomal dysfunctions resulting in autophagic and lysosomal acidification and biogenesis impairment appear to be hallmarks of many inherited and acquired neurodegenerative diseases. Lysosomes are not yet seen as a sort of cellular dump but are crucial in determining key signalling paths and processes involved in the clearance of aggregated proteins. Thus, the possibility of pharmacologically modulating them deserves great interest. Despite the potentiality of metformin in this context, many additional important issues, such as dosing, should be addressed in the future.

## 1. Introduction

Metformin (1,1-dimethylbiguanide hydrochloride) is one of the most employed drugs but has ancient origins, as efficaciously narrated by Bayley [1]. Briefly, the therapeutical employment of this type of molecule comes from the experience with an herbal ancestor, the *Galega officinalis* (*G. officinalis*), a herbaceous plant belonging to the Fabaceae family. In 1772, the employment of the *G. officinalis* was suggested to treat intense thirst and frequent urination that now we know to be the classic symptoms of diabetes mellitus. In 1800, the chemical analysis of *G. officinalis* revealed that it is enriched in guanidine and derivatives. In 1918, guanidine was demonstrated to reduce glycaemia in animals. However, the way that led to metformin synthesis was taken from re-evaluating a guanidine derivative (proguanil) used against malaria and found to reduce glycaemia. The chemical modification of proguanil led to the onset of a molecule chemically identic to metformin, in 1949, which was originally used to treat influenza and called “flumamine”. Only in 1957 was metformin proposed against diabetes mellitus by J Sterne [1]. Currently, metformin is recommended in the first tier against type 2 diabetes mellitus when insulin can be avoided [2]. Other biguanides were tested during the last century, but their high risk of developing lactic acidosis discouraged employment [1]. 

The success of metformin in type 2 diabetes mellitus treatment is explained by its effectiveness in maintaining good glycaemic control over time [2], its low cost and the absence of significant side effects, except nausea and diarrhoea [2], and the possibility of developing vitamin B12 deficiency [3]. Vitamin B12 deficiency has been described especially in patients taking metformin for a long time. It seems to be caused by the action of metformin on its absorption. In particular, metformin could reduce the calcium-dependent interaction between the intrinsic factor–vitamin B12 complex and the ileal cubilin receptor, which mediates the subsequent endocytosis of vitamin B12. Other mechanisms, such as reduced intrinsic factor synthesis, are under investigation [4]. Metformin is orally taken (around 1–2 g daily or 20 mg/kg daily) and is primarily adsorbed in the small intestine (around 50–60%), ranging in plasma concentrations near 10–40 μmol/L [5]. Very discordant data regarding the “therapeutic” metformin concentrations in both in vitro and in vivo studies have been reported in the literature [5]. Metformin is not catabolised and is excreted by the kidney [6].

Through analyses performed in humans using ^11^C-metformin positron emission tomography–computed tomography (PET-CT), the intestine, liver, and kidneys were reported to be the main organs where metformin accumulates with a small muscle contribution [7]. In particular, the liver has been described as the main organ where the glucostatic metformin action occurs. Currently, the involvement of the gastrointestinal system, including gut microbiota, is being evaluated [8].

At the subcellular level, mitochondria have been considered the unique target organelle of metformin action for a long time. Mitochondrial metformin’s main targets are the respiratory complex I and glycerol-3-phosphate dehydrogenase (GPD2) [9]. Recently, other targets of action, including some lysosomal pathways, have been identified [9,10,11,12].

Different possible biological properties of metformin are under investigation. In particular, many studies are trying to clarify if metformin might be an anti-ageing [13], anti-inflammatory [14], anti-oxidative agent [15], and an autophagic inductor [16]. Moreover, the metformin action on epigenomics and micro-RNAs has been described. Metformin could modulate many epigenetic enzymes, such as histone acetyltransferase, class II histone deacetylases, and DNA methyltransferases [17]. The attempt to repurpose metformin for additional diseases other than type 2 diabetes mellitus is ongoing; in particular, type I diabetes mellitus, polycystic ovary syndrome, cancer, neurodegenerative, cardiovascular and renal diseases, and infective diseases like COVID-19 are being considered [6,18]. The possible benefits identified in these disorders are likely not only due to the glycaemic control prompted by metformin; thus, it should be essential to characterise better all its cellular and molecular targets. Based on these premises, this review specifically focuses on discussing the action of metformin on lysosomal-dependent cellular processes and lysosomal targets in the view of a neuroprotective therapeutic approach.

## 2. Metformin Modulates Lysosomal-Dependent Processes

Lysosomes are organelles primarily responsible for the catabolic degradation and recycling of macromolecules. The low pH of the lumen is warranted by the vacuolar ATPase (v-ATPase) and allows the catalytic action of around 60 acid hydrolases. In addition, the lysosomal protein set includes luminal enzyme activators, protective and transport factors, and, at the membrane, ion channels, transporters, the lysosome-associated membrane proteins (LAMPs), and soluble N-ethylmaleimide-sensitive factor attachment protein receptors (SNARE) proteins that modulate the fusion of vesicles or other organelles. In addition to their degradative function, lysosomes have emerged as key organelles involved in cell signalling and homeostasis. They are dynamic structures that vary in composition, number, size, and shape, along with cellular conditions such as stress and nutrient enrichment. The release of intra-lysosomal calcium modulates the fusion of lysosomes with autophagosomes, endosomes, and endoplasmic reticulum [19] and signal transduction [20].

The lysosomal membrane is involved in the mechanistic target of rapamycin complex 1 (mTORC1) activation and, thus, participates in the cellular response to nutrient and growth factor deprivation. Small GTPases, Rag GTPases, and Ras homolog enriched in brain (RHEB) GTPases mediate the interaction and activation of mTORC1 at the lysosome [20]. Lysosomal cholesterol accumulation can also induce mTORC1 recruitment to the lysosome [21]. Lysosomes are responsible for the health of other organelles, such as endoplasmic reticulum and mitochondria, that are renewed by autophagy. In turn, endoplasmic reticulum stress and mitochondrial signals modulate autophagy [20]. As reviewed by Settembre et al., lysosomal dysfunctions are related to neurodegeneration and protein aggregation, skeletal muscle and heart impairment, bone, cartilage, kidney, and eye alterations, response to infection, and tissue development [20].

Metformin modulates several cellular processes that involve lysosomes. First, evidence has been collected demonstrating that metformin modulates autophagy, a lysosomal-dependent process of cellular clearance. Three types of autophagy have been identified: the macro-autophagy, the micro-autophagy, and chaperone-mediated autophagy (CMA). All these forms include a degradation step performed by lysosomes. Thus, it is easily understandable why lysosomal dysfunctions typically impair the autophagic processes and all autophagy-dependent events. Macro-autophagy is the most common type: it acts through the formation of vesicles, called autophagosomes, which enclose cytoplasmatic components and fuse with lysosomes to form autolysosomes. Cargo degradation occurs in autolysosomes. During micro-autophagy, lysosomes internalise directly the cytosolic material. CMA takes its denomination from the involvement of chaperone proteins. They form a complex with proteins addressed to degradation and interact with LAMP-2A, allowing the lysosomal internalisation of targeted proteins. Autophagy is modulated by numerous signalling pathways, such as the target of rapamycin (TOR) kinase, and upstream pathways, such as adenosine 5′-monophosphate (AMP)-activated protein kinase (AMPK) and the hypoxia-inducible factor (HIF) [22]. Nutrient deprivation and hypoxia induce autophagy to favour metabolic recycling. Autophagy is important for mitochondria renewal and for reducing oxidative stress. This process is specifically referred to as “mitophagy”. Moreover, autophagy reduces the formation of protein aggregates involved in neurodegeneration, such as α-synuclein, tau, and mutant huntingtin aggregates [22]. In this view, autophagy inducers are evaluated to prevent or treat neurodegenerative diseases.

Regarding metformin and autophagy, both inducing and inhibitory actions have been identified with a prevalence of the first ones, possibly depending on the interaction with the pre-existent activation/deregulation of signalling pathways [16]. 

Metformin accelerates autophagic flux in vascular smooth muscle cells (VSMCs), increasing autophagosome–lysosome fusion, LAMP-1 expression, and feasibly lysosomal function. This reduces the autophagy and mitophagy dysfunctions that are reported to occur in type 2 diabetes mellitus patients (i.e., p53, p21, p16 proteins, and senescence-associated β galactosidase) and senescence-associated secretory phenotype (SASP) (i.e., matrix metalloproteinase 2 (MMP2), interleukin 6 (IL-6), and transforming growth factor (TGF) β) and increases the proliferation and migration rate of senescent VSMCs [23]. Remaining in the cardiovascular context, metformin reactivates autophagic flux in cardiomyocytes after the treatment with crizotinib (a tyrosine kinase inhibitor used in anaplastic lymphoma kinase (ALK)-positive metastatic non-small cell lung cancers), which induces high cardiotoxicity also through the block of autophagosome–lysosome fusion [24]. Metformin protects against the cardiotoxicity induced by another anticancer drug, doxorubicin. Using FVB/N mice and H9C2 cardiac myoblasts, a reduction in autophagy and mitophagy was detected after metformin–doxorubicin treatment [25]. Metformin reduces atherogenesis in high-fat diet-induced apolipoprotein E deficient (apoeE-/- mice, promoting Krueppel-like factor (KLF2)-mediated autophagy [26].

Metformin promotes CMA: this action decreases amyloid β deposition in the brain of amyloid precursor protein (APP)/presenilin 1 (PS1) mouse model of Alzheimer’s disease [27]. Another paper demonstrated that metformin increases autophagy in microglial cells, promoting the phagocytosis of amyloid β and tau proteins in APP/PS1 mice [28].

Further, metformin ameliorates the autophagic impairment after spinal cord injury induced in rats, promoting autophagosome–lysosome fusion, a step demonstrated to be helpful for microglial cell activation and myelin debris clearance [29]. However, another report proved that metformin increases the accumulation of autophagosomes but also γ-secretase activity and then amyloid β formation [30].

Metformin rescues autophagy and cell viability in fibroblasts isolated from patients affected by UDP-N-acetylglucosamine 2-epimerase/N-acetylmannosammine kinase (GNE) myopathy, an autosomal recessive disease [31].

In addition, metformin reduces the Th17 inflammatory cytokine profile, enhancing autophagy [32].

Autophagy and mitophagy dysfunctions are reported to occur in type 2 diabetes mellitus patients and β-cells of type 2 diabetic mice. These dysfunctions are supposed to be involved in the pathophysiology of the disease [33]. Metformin reverses mitophagy dysfunctions in peripheral blood mononuclear cells (PBMCs) isolated from type 2 diabetes mellitus patients, originally showing decreased expression of phosphatase and tensin homolog (PTEN)-induced kinase 1 (PINK1) and parkin [34] and promotes a neuroprotective effect in hyperglycaemia associated with cerebral ischaemia/reperfusion injury modulating mitophagy and apoptosis [35]. The system PINK1/Parkin is the main activating path of mitophagy [36].

Many physiological and pathological events, such as senescence, the removal of unfolded and amyloidogenic proteins, inflammation, cardiovascular and nervous system protection, and anti-tumorigenesis, could be regulated by modulating autophagy with metformin treatment. Metformin can regulate autophagy through many different signalling pathways, including AMPK-related pathways, regulated in development and DNA damage responses 1 (Redd1)/mammalian target of rapamycin (mTOR), signal transducer and activator of transcription (STAT), sirtuin (SIRT), Na^+^/H^+^ exchangers (NHEs), mitogen-activated kinase (MAPK), prokineticin 2 (PK2)/PK receptor (PKR)/protein kinase B (AKT)/glycogen synthase kinase 3β (GSK3β), and tribble pseudokinase 3 (TRIB3) [16].

As previously indicated, metformin can exert a bidirectional role in autophagy, decreasing the process extent in some conditions. AMPK/nuclear factor kappa-light-chain enhancer of activated B cells (NF-kB), Hedgehog, some miRNAs such as miR-570-3p and miR-142-3p, phosphoinositide 3-kinase (PI3K)/AKT, mTOR, and metformin-mediated reduction in endoplasmic reticulum stress have been described as pathways involved in metformin inhibition of autophagy. It was hypothesised that the final effect of metformin on autophagy could be dependent on metabolic/molecular differences in different organs/cells and pre-existing activation/deregulation of signalling pathways. This is of particular importance when metformin is employed in cancer cells [16]. 

In Table 1, we collected data reported in the scientific literature describing the modulatory effect of metformin in in vitro and in vivo models used for some neurodegenerative diseases. It appears evident that, in this context, metformin promotes autophagy in the large majority of studies. Moreover, AMPK activation emerged as the main pathway investigated and involved in this process. 

Metformin has been described to modulate the content of the lysosomal proteins, cathepsins. Cathepsin D level was increased after metformin treatment, together with autophagy, in a δ-sarcoglycan-deficient mouse model of dilated cardiomyopathy [45]. Moreover, the anti-photoageing effect of metformin was mainly ascribed to increased autophagic flux by inducing cathepsin D [46]. 

It was speculated that metformin could modulate the endocytic cycle by changing endosomal pH through endosomal NHEs or v-ATPase [47]. 

## 3. Lysosomal Targets Recognised by Metformin

Metformin has been demonstrated to recognise different lysosomal molecular targets. The recruitment of some of these targets leads to AMPK activation through a lysosomal path.

The endosomal NHEs were found to be possible targets of metformin in *Caenorhabditis elegans* (*C. elegans*) (NHX-5) and *Drosophila melanogaster* (NHE3) and to be involved in metformin-promoted regulation of the endocytic cycle [11,47]. Mammalian NHEs are integrally inserted in plasma membranes (NHE1-5) or organelles (NHE6-9) and allow the exchange of H^+^ with Na^+^ or K^+^. So far, nine forms with different tissue expressions and functional features have been recognised [48]. Among them, NHE6 and NHE9 were mainly identified in endosomes [48]. NHE6 has been involved in some cases of Angelman syndrome and NHE9 in other neurological disorders such as autism [48]. Mutations in the gene coding for NHE6 (*SLC9A6*) have been related to Christianson disease, characterised by neurological impairment, and NHE6-null neurons show impaired lysosomal functionality associated with altered endosome trafficking [49]. In addition, cortical neurons produced from NHE6 knock-out human-induced pluripotent stem cells show lysosomal and autophagic defects along with increased phosphorylated and insoluble tau [50]. To the best of our knowledge, no studies have been specifically addressed to demonstrate the direct action of metformin on NHE6; thus, it could be currently only speculated based on data obtained in *C. elegans* and *Drosophila melanogaster*.

A lysosomal pathway promoted by metformin and leading to AMPK activation was described by Zhang et al. [10]. The authors demonstrated that AMPK activation depends on the expression of axin, Lamtor1, i.e., a component of Ragulator, and the v0c subunit of v-ATPase. This mechanism of AMPK activation is regulated by cellular energy level and depends on a macromolecular complex formation on the lysosomal membrane [10,47]. 

Ragulator is a protein complex localised on late endosomes and lysosome membranes which works as a signalling platform. The complex is composed of five Lamtor subunits and is closely associated with RagA/B and RagC/D GTPases, v-ATPase (responsible for proton transport across membranes and acidification of lysosomal lumen), and SLC38A9, a neutral amino acid transporter [51]. Moreover, Ragulator can interact with axin, a scaffold protein for liver kinase B1 (LKB1). When the cellular energy level is low, as in starvation conditions, axin can interact with Ragulator and recruit LKB1 to the complex. LKB1 activates AMPK, anchored to the lysosomal membrane by a myristoyl tail. On the contrary, when nutrients are available and cell energy is high, axin is not able to interact with Ragulator, and AMPK is not active. In this condition, mTORC1 binds to Ragulator and promotes anabolic signalling [51,52].

Using mice with no expression of axin or Lamtor1 in the liver, Zhang et al. demonstrated that the activation of AMPK by metformin requires these proteins [10]. Furthermore, experiments in HEK293T cells knocked down for the v0c subunit of v-ATPase showed that the translocation of axin/LKB1 on lysosomes was suppressed, and metformin could not activate AMPK [10,47].

The assembly of v-ATPase-Ragulator-Axin/LKB1-AMPK complex in response to metformin was studied in mouse embryonic fibroblasts (MEFs), and it was found that the v-ATPase could be the target and the binding site of metformin. Stimulating the formation of the complex, metformin activates AMPK and, at the same time, inhibits mTORC1 [10].

Recently, it has been demonstrated that metformin can interact with the protein presenilin enhancer 2 (PEN2) on the lysosome surface [12]. PEN2 is a regulatory subunit of γ-secretase, a transmembrane proteolytic complex involved in the production of amyloid β-peptide in Alzheimer’s disease [53].

A lysosomal protein extract from MEFs was screened with photoactivable metformin probes using affinity-based techniques to identify metformin-binding proteins. Among 113 possible partners individuated, shRNA silencing experiments allowed the identification of PEN2 as a metformin binding target [12,54,55].

Mass spectrometry analysis and in silico modelling showed that metformin binds the cytosolic N-terminal domain of PEN2; in particular, three amino acid residues of PEN2 seem to be essential for the interaction with the protein: tyrosine 47, phenylalanine 45, and glutamate 40. Mutations of these residues prevent interaction between metformin and PEN2 [12,54].

**Figure 1:**
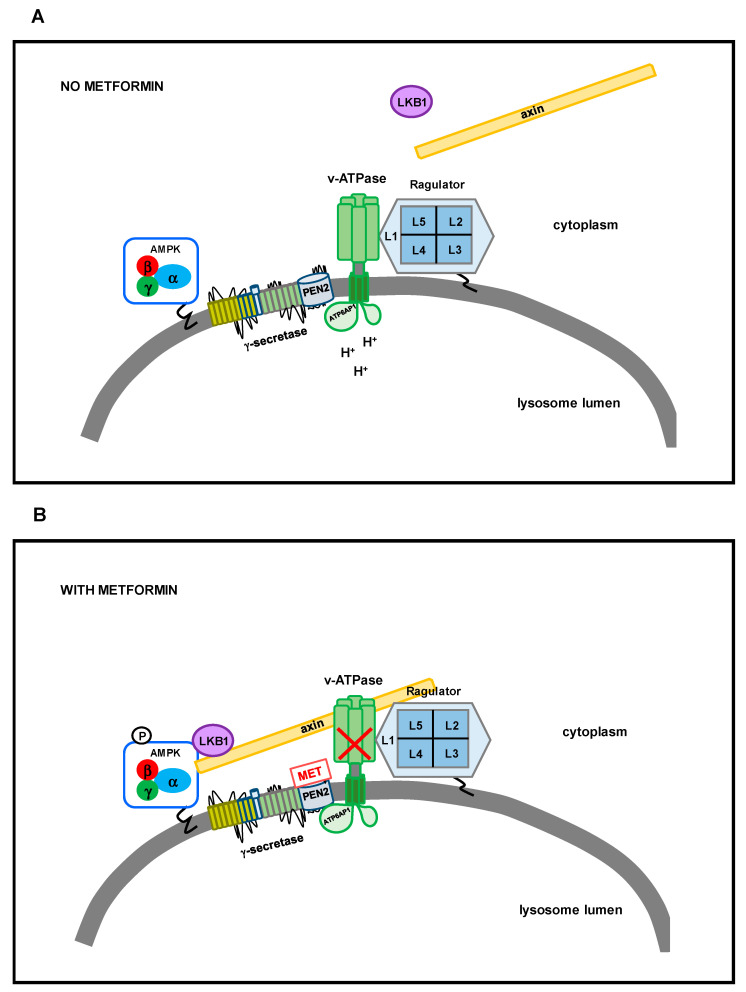
A lysosomal signalling pathway of AMPK activation by metformin. An intricate hub of signalling proteins resides on the lysosome membrane; among them, the Ragulator complex, v-ATPase, γ-secretase, and AMPK can interact with each other, regulating lysosome response to stimuli (**A**). Metformin binds to PEN2, a subunit of γ-secretase. The metformin-PEN2 complex joins with a cytoplasmic subunit (ATP6AP1) of v-ATPase and inhibits the proton pump. In this condition, axin associates with Ragulator and v-ATPase and recruits LKB1, which activates AMPK by phosphorylation (**B**). L1-5: Lamtor1-5; MET: metformin.

Next, it has been shown that metformin-bound PEN2 joins with ATP6AP1, a luminal domain of the v0 subunit of v-ATPase, causing v-ATPase inhibition and enrolment of axin to v-ATPase-Ragulator complex [12,56]. Axin recruits LKB1 and elicits AMPK activation through a lysosomal pathway without changing AMP/ATP levels [10,12]. This mechanism of AMPK activation was confirmed using PEN2 and ATP6AP1-depleted MEF cells [12] (Figure 1).

This metformin action is triggered by low doses (pharmacologically relevant) of the drug (5–30 μM) and seems to be involved in lifespan extension in *C. elegans* [12,54]. Moreover, in murine models, the glucose-lowering effect and the reduction in hepatic triglyceride content appeared to be linked to this mechanism [12,54]. At last, using bioinformatic approaches, it has been suggested that, at low concentrations, the antiparasitic effect of metformin against *Trichinella spiralis* (*T. spiralis*) may involve the PEN2-ATP6AP1 axis [57].

The transcription factor EB (TFEB), a member of the microphthalmia-transcription factor E (MiTF/TFE) family of transcription factors, plays a crucial role in autophagy and lysosome biogenesis and functions. TFEB is phosphorylated by mTOR in normal conditions and localises in the cytoplasm. Conversely, under stress conditions, TFEB is dephosphorylated and moves to the nucleus, activating target gene transcription [58].

In 2019, Wu et al. demonstrated that metformin promotes random-pattern skin flap survival via a TFEB-dependent mechanism [59]. Metformin treatment decreases oxidative stress and apoptosis and stimulates angiogenesis, promoting skin flap vitality in a model of random skin flaps. The underlying mechanism of these effects was found to be the activation of autophagy via TFEB nuclear translocation [59].

In a mouse model of non-alcoholic fatty liver disease (NAFLD), it has been demonstrated that metformin decreases hepatic steatosis and insulin resistance, promoting TFEB-dependent autophagy [60]. Metformin treatment promotes the nuclear translocation of TFEB in the liver of NAFLD mice [60]. If *TFEB* expression is knocked down, metformin cannot activate autophagy, showing that metformin induces autophagy via TFEB activation [60].

Recently, it has been demonstrated that metformin decreases hepatic lipid accumulation and liver injury in mouse models of high-fat diet-induced liver disease (HFD) and NAFLD via activation of tristetraprolin (TPP), a mRNA binding protein [61]. Moreover, this study showed that metformin promotes autophagy in primary hepatocytes, increasing TFEB nuclear translocation in a TPP-dependent manner [61].

Asparaginyl endopeptidase or legumain is a cysteine protease localised in endosomes and lysosomes, which hydrolyses specifically asparaginyl bonds in non-terminal regions of polypeptides [62]. The *LGMN* gene, mapped on chromosome 14, encodes this enzyme, which is involved in various physiological processes regulation, such as bone remodelling, kidney function, and hematopoietic and immune system functions. Moreover, it seems to be implicated in different diseases, like cancers, neurodegenerative and cardiovascular diseases [62]. Recently, Tu et al. [63] found that metformin suppresses cancer progression in choriocarcinoma, downregulating legumain expression. In particular, metformin treatment decreased legumain mRNA and protein expression in the choriocarcinoma cell lines JAR and JEG-3, inducing autophagy in a legumain-dependent manner [63].

## 4. Metformin and Neurodegenerative Diseases

Studies reporting the potential benefits of metformin in some neurodegenerative diseases, such as Alzheimer’s, Parkinson’s, and Huntington’s diseases, were recently reviewed by Rotermund et al. [64] and Du et al. [65]. However, the results are sometimes contradictory, and the molecular and biological mechanisms are unclear. Metformin can cross the blood–brain barrier and directly act on the central nervous system [66]. As recently reviewed by Cao et al., metformin could modulate the onset and progression of neurodegenerative disorders by acting on several common cues that are associated with cognitive impairment and neurodegeneration: (a) impaired glycemic control; (b) chronic inflammation; and (c) AMPK and downstream signalling pathway deregulation. In addition, metformin can produce (a) direct effects on microglia with the drive toward the M2 phenotype polarisation; (b) effects on gut microbiota; (c) induction of brain-derived neurotrophic factor (BDNF) and glial cell line-derived neurotrophic factor (GDNF) expression; and (d) effects on neurogenesis and neurotransmission system [66]. However, examining systematic reviews exploring the effects of metformin on the prevention and progression of some neurodegenerative diseases, the conclusions are conflicting. The meta-analysis of clinical observational studies performed by Luo et al. regarding a possible correlation between metformin and Alzheimer’s disease risk indicates that metformin does not reduce the risk of developing the disease but conversely increases it in Asians [67]. Similar conclusions were gained by the meta-analysis dated 2022, performed by Malazy et al., who did not recognise the protective role of metformin against dementia [68]. The systematic review elaborated by Munoz-Jimenez et al. regarding the effects of metformin and other antidiabetic drugs in Alzheimer’s disease patients recognised promising improvements in cognitive performance, but they were not sufficient to recommend these treatments with the current information [69]. On the other hand, the meta-analysis performed by Campbell et al. proved the reduction in dementia onset among diabetic patients treated with metformin compared to others and speculated that vitamin B12 deficiency, which is caused by the chronic assumption of metformin could be the reason for some contradictory evidence [70]. It is questionable if possible benefits triggered by metformin could be restrained to the diabetic population.

Similar conclusions have been reached by recent meta-analyses performed to evaluate the correlation between metformin and the risk of developing Parkinson’s disease: both the papers by Xie et al. [71] and Qin et al. [72] failed to determine a positive association based on the studies examined. 

Thus, the interest in metformin in this field is currently high because of data achieved using cellular or animal models and demonstrating that this drug interferes with processes involved in the most frequent neurodegenerative diseases. However, it should be pivotal to explore better the molecular mechanisms elicited by metformin and understand if and how they could be beneficial in pathological contexts different from diabetes.

## 5. Why Metformin Lysosomal Targeting Could Be Helpful in Neurodegenerative Diseases

Given the importance of lysosomal dysfunction in neurodegeneration and in the onset and progression of many neurodegenerative diseases, the investigation of metformin lysosomal targeting could be relevant. Inherited disorders classified as “lysosomal storage diseases” are often characterised by devasting effects on the nervous system [73], and lysosomal dysfunctions are commonly identified in adult neurodegenerative diseases [74,75]. An emblematic case is constituted by Niemann–Pick type C disease, an inherited lysosomal disorder that shares many similarities with Alzheimer’s disease, such as amyloid β deposition and neurofibrillary tangles [76].

As previously discussed, for many years, lysosomes have been considered organelles involved in the catabolism and degradation of molecules destined for elimination. Their function is now re-evaluated and enlarged to processes such as endocytosis, exocytosis, energetic metabolism, signalling modulation, and autophagy [77]. 

As reviewed by Cui et al., autophagic dysfunction has been observed in Alzheimer’s disease, where it is involved in determining amyloid β and tau-altered turnover and deposition, Parkinson’s disease with a correlation with α-synuclein accumulation, and Huntington’s disease [78]. Approaches mired to re-establish autophagy have been related to amyloid β reduction [79,80]. Similarly, neurodegeneration was decreased in mouse models of tauopathy after autophagic induction [81]. In addition, it was demonstrated that rapamycin treatment in murine models of Alzheimer’s disease reduced the exosomal release of amyloid β and tau proteins [82]. Rapamycin decreased mutant huntingtin aggregation in the brain of *Drosophila melanogaster* [83].

Thus, autophagic induction appears to be a promising tool to be explored, and different therapeutic strategies are being tested to this end in neurodegenerative diseases.

Further, alterations of luminal endosomal pH can compromise endosome maturation and function. Dysfunctions of endosomal–lysosomal pH seem to be involved in different neurodegenerative diseases such as Alzheimer’s and Parkinson’s diseases and amyotrophic lateral sclerosis (ALS) [84]. The TFEB-v-ATPase path was demonstrated to be involved in modulating microglia and immune response in Alzheimer’s disease and tauopathy through its effects on lysosomes [85]. Defective presenilin 1 was related to v-ATPase impairment, autophagic and lysosomal acidification impairments, and deregulated calcium homeostasis [86]. Significantly, loss-of-function mutations in the *PSEN1* gene, encoding presenilin 1, lead to early-onset familial Alzheimer’s disease [87]. 

Mutations in lysosomal proteins or modulators of lysosomal functionality have been reported in frontotemporal dementia and ALS [75]. A common genetic cause of these diseases is the expansion of the *C90RF72* gene, which determines the production of repeat-associated non-AUG (RAN) proteins [88]. RAN proteins are neurotoxic because they alter different mechanisms, such as autophagy and lysosomal functionality. Approaches mired to reduce RAN protein levels are one of the emerging therapeutic strategies for these pathologies [89]. Recently, it has been demonstrated that metformin inhibits RAN protein translation in *C9orf72*-ALS BAC mice [90]. 

Currently, two papers analysed the effects of metformin treatment in lysosomal storage diseases. Cholesterol accumulation is a feature of Niemann–Pick disease [91]. One of these papers reported the effects of metformin plus 2-hydroxypropyl-β-cyclodextrin co-treatment on a Niemann–Pick murine model (*Npc1*^−/−^ mouse). The authors did not identify an improvement in survival or a decrease in cholesterol. However, they reported that metformin decreased the inflammatory response and the release of proinflammatory cytokines in the brain, spleen, and liver [92]. The second paper described the effects of metformin treatment in a cellular model of metachromatic leukodystrophy. Human Schwann cells were isolated from the spinal nerve, and the CRISPR/Cas9 approach was employed to modify the *ARSA* gene. Metformin treatment ameliorated the impairment of mitochondrial respiration and reduced the release of reactive oxygen species (ROS) [93].

## 6. Concluding Remarks

Considering clinical efficacy and cost-effectiveness, metformin is a low-cost and generally well-tolerated drug with multiple beneficial effects in addition to blood glucose lowering [94]. Its employment has been explored in many other diseases, in addition to type 2 diabetes mellitus, from cardiovascular to neurodegenerative diseases and cancer. An article defines metformin as “the aspirin of the 21st century” [95]. 

However, many cues remain to be better investigated. Metformin plays pleiotropic effects and targets multiple organelles and systems/apparatus [47,96]. The impact played by metformin on lysosomal processes, such as autophagy and organelle acidification, and on lysosomal targets previously discussed, appears promising to consider this treatment in the context of neurodegenerative diseases. Given the importance of lysosomal dysfunctions in neurodegenerative diseases, the possibility of modulating them deserves interest.

**Figure 2:**
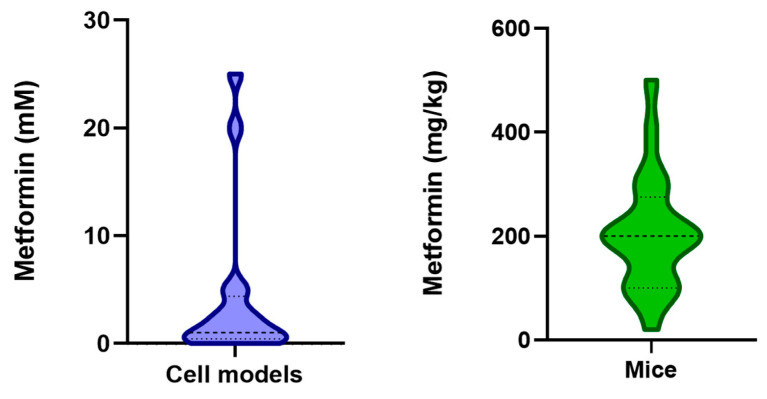
Representation through violin plots of metformin doses employed in cellular and murine models used to explore the effects related to neurodegeneration. The duration of treatment was significantly different among the studies; also, the modality of metformin administration to mice varied (orally, intra-peritoneally, intra-gastric, etc.). The search was carried out using Pubmed, selecting papers published in the last 5 years, with the keywords “metformin AND neurodegenerative disease AND cell AND mouse” [27,37,38,40,90,92,100,101,102,103,104,105,106,107,108,109,110,111,112,113,114,115,116,117,118,119,120,121,122].

As underlined, contradictory results have been collected about the efficacy of metformin in preventing or alleviating neurodegenerative diseases; thus, it is difficult to define if and possibly how metformin could be proposed apart from the context of diabetes. An important issue appears to be the metformin concentration used in studies (Figure 2). There is great variability, and supraphysiological doses are often used in animal or cellular models, which are also 10–100 times higher than those found in diabetic patients [97]. Currently, metformin is orally administered, and after the uptake by the liver, its mean circulating concentration is 10–40 μM in animals and humans [97]. However, in murine models, it was found that metformin is retained in some tissues in concentrations higher than those identified in the plasma [98]. Thus, it could be complicated to establish the specific molecular mechanisms involved and the correct dose to elicit the processes needed. Accordingly, tissue redistribution and retaining are also important.

In addition, the effects due to chronic metformin intake should be considered. It is well known that a correlation between the chronic intake of metformin and vitamin B12 deficiency in diabetic patients exists due to the impairment in vitamin B12 absorption metformin caused [4]. Vitamin B12 deficiency significantly impacts the functionality of different organs/apparatus, including the nervous system [99].

Thus, we can conclude that a better exploration of metformin action on lysosomal behaviour and the modality (dose, administration, etc.) needed to exploit these effects could help to consider the possibility of therapeutic repurposing in neurodegenerative diseases.

## Figures and Tables

**Table 1:** Reported effects on autophagy in in vitro and in vivo models used for some neurodegenerative diseases. The search was performed in Pubmed (keywords: metformin, autophagy, and neurodegenerative), only considering experimental papers.

Cell/Animal Model	Disease	Autophagy Induction	Signalling Pathway Involved	References
293THK cells and male transgenic APP/PS1 (C57BL/6) mice	Alzheimer’s disease	↑	TGF-β activated kinase 1 (TAK1)-Ikappa B kinase (IKK)α/β-heat shock cognate protein (Hsc) 70	[27]
Paraquat-treated SH-SY5Y cells and C57BL6 mice	Parkinson’s disease	↑	AMPK	[37]
RML prion-infected ScCAD5 cells	Prion disease	↑	Not reported	[38]
Adult male Wistar rats subjected to transient forebrain global ischaemia	Cerebral ischaemia	↑	AMPK	[39]
Primary cerebellar granule neurons and SH-SY-5Y cells	Alzheimer’s disease	= or ↓	AMPK	[40]
Organotypic cultures of spiral ganglion neurons isolated from cadmium-treated C57BL/6 mice	Cadmium-induced spiral ganglion neuron degeneration	↑	Not reported	[41]
db/db mice and high glucose-cultured HT22 cells	Diabetes-induced tau hyperphosphorylation	↑	AMPK	[42]
*Caenorhabditis elegans*	Huntington’s disease	↑	AMPK	[43]
Induced pluripotent stem cell-derived astrocytes with *POLG* mutations	Mitochondrial diseases	↑	AMPK	[44]
